# Identification of the Expression Patterns and Potential Prognostic Role of 5-Methylcytosine Regulators in Hepatocellular Carcinoma

**DOI:** 10.3389/fcell.2022.842220

**Published:** 2022-02-16

**Authors:** Yong Liu, Shunzhen Zheng, Tao Wang, Ziqi Fang, Junjie Kong, Jun Liu

**Affiliations:** ^1^ Department of Liver Transplantation and Hepatobiliary Surgery, Shandong Provincial Hospital, Cheeloo College of Medicine, Shandong University, Jinan, China; ^2^ Department of Liver Transplantation and Hepatobiliary Surgery, Shandong Provincial Hospital Affiliated to Shandong First Medical University, Jinan, China; ^3^ Department of Clinical Laboratory, Shandong Provincial Hospital, Cheeloo College of Medicine, Shandong University, Jinan, China

**Keywords:** 5-Methylcytosine, hepatocellular carcinoma, tumor immune microenvironment, m5Cscore, prognosis

## Abstract

**Background:** Hepatocellular carcinoma (HCC) is the most common primary liver cancer with a poor prognosis. 5-methylcytosine (m5C) modification plays a nonnegligible role in tumor pathogenesis and progression. However, little is known about the role of m5C regulators in HCC.

**Methods:** Based on 9 m5C regulators, the m5C modification patterns of HCC samples extracted from public databases were systematically evaluated and correlated with tumor immune and prognosis characteristics. An integrated model called the “m5Cscore” was constructed using principal component analysis, and its prognostic value was evaluated.

**Results:** Almost all m5C regulators were differentially expressed between HCC and normal tissues. Through unsupervised clustering, three different m5Cclusters were ultimately uncovered; these clusters were characterized by differences in prognosis, immune cell infiltration, and pathway signatures. The m5Cscore was constructed to quantify the m5C modifications of individual patients. Subsequent analysis revealed that the m5Cscore was an independent prognostic factor of HCC and could be a novel indicator to predict the prognosis of HCC.

**Conclusion:** This study comprehensively explored and systematically profiled the features of m5C modification in HCC. m5C modification patterns play a crucial role in the tumor immune microenvironment (TIME) and prognosis of HCC. The m5Cscore provides a more holistic understanding of m5C modification in HCC and provides a practical tool for predicting the prognosis of HCC. This study will help clinicians identify effective indicators of HCC to improve the poor prognosis of this disease.

## Introduction

Hepatocellular carcinoma (HCC) is the most common primary liver cancer and is currently the third leading cause of cancer-related death worldwide (Bray et al., 2018; Islami et al., 2021). Although effective measures such as HBV vaccine immunization and health education have been implemented, the incidence of HCC has increased from 46.3 per 100,000 to 62.8 per 100,000 between 2005 and 2014 (Bosetti et al., 2014; Sayiner et al., 2017; Hester et al., 2020). Despite improvements in surveillance and treatment, the prognosis of HCC remains poor and the median overall survival (OS) time is only approximately 6–2 months once diagnosed (Bosetti et al., 2014). Prognostic assessment is a crucial step in the management of patients with HCC. An increased concentration of α-fetoprotein (AFP) is associated with poorer prognosis. Disappointingly, relevant studies reported that its sensitivity was approximately 60%, and more worryingly, its specificity was 80% (Marrero et al., 2009; Lok et al., 2010). Other tumor markers, such as angiopoietin 2 or vascular endothelial growth factor, might refine prognostic prediction in statistical modeling, but cannot yet be incorporated into the individual assessment of a specific patient (Forner et al., 2018). Therefore, searching for effective biomarkers for predicting the prognosis of HCC and developing novel targets for HCC treatment are urgent.

RNA modifications, which are more than 150 types of modifications reported thus far, are prevalent posttranscriptional modifications and play a critical role in regulating biological processes (Roundtree et al., 2017; Boccaletto et al., 2018). 5-methylcytosine (m5C), an important posttranscriptional modification, is present in diverse RNA species and participates in many aspects of gene expression, including RNA export, ribosome assembly, translation, and RNA stability (Trixl and Lusser, 2019). Increasing evidence has suggested that m5C modification plays a nonnegligible role in tumor pathogenesis and progression. It was reported that the m5C regulator YBX1 maintained the mRNA stability of the oncogenic gene heparin binding growth factor (HDGF) by binding to m5C methylated sites and recruiting ELAVL1, thus exerting an oncogenic role in bladder cancer development through the activation of HDGF (Chen et al., 2019). In addition, Gao et al. (Gao et al., 2019) found that high expression of NSUN2 could promote the proliferation and tumorigenesis of gallbladder carcinoma cells both *in vitro* and *in vivo* by closely cooperating with ribosomal protein L6. Recently, the role of m5C modification in cancers, including lung cancer, colon carcinoma, bladder cancer, and thyroid carcinoma, has been explored (Pan et al., 2021a; Pan et al., 2021b; Chen et al., 2021; Gao et al., 2021; Geng et al., 2021; Gu et al., 2021; Hu et al., 2021; Li et al., 2021; Liu et al., 2021; Xu et al., 2021). In addition, He et al. (He et al., 2020a) built a gene signature including 2 m5C regulators and found that it could effectively predict the prognosis of HCC. However, the signature is limited to the number of m5C regulators, while their role in the pathogenesis and progression of HCC depends on the interaction among the multiple m5C regulators.

In this study, we systematically evaluated the m5C modification pattern and tumor immune microenvironment (TIME) in HCC patients. We revealed three distinct m5C modification patterns in HCC; these clusters were characterized by differences in prognosis, immune cell infiltration, and pathway signatures. Based on the m5C regulators and related genes, a model (termed “m5Cscore”) was constructed to quantify the m5C modification patterns of individual patients. The study also demonstrated that the m5Cscore could serve as a practical tool to predict the prognosis of HCC.

## Materials and Methods

### Data Extraction and Preprocessing

The RNA sequencing data and relevant clinicopathological features of 374 HCC and 50 normal samples were obtained from The Cancer Genome Atlas database (TCGA; https://portal.gdc.cancer.gov/). Gene expression data (measured in fragments per kilobase of exon per million fragments mapped or FPKM) were transformed into transcripts per kilobase million (TPM). Somatic mutation data were extracted from the TCGA data portal. Furthermore, an eligible HCC set was downloaded from the International Cancer Genome Consortium database (ICGC; https://icgc.org/) and served as the validation cohort. All human HCC tissue samples used in this study were obtained from patients who underwent surgery in the Shandong Provincial Hospital Affiliated to Shandong First Medical University. This project was approved by the Ethics Committee of Shandong Provincial Hospital Affiliated to Shandong First Medical University and was performed in accordance with the Declaration of Helsinki. Each participant provided written informed consent.

### The Landscape of m5C Regulators in Hepatocellular Carcinoma

A total of 10 m5C regulators were obtained and curated from previous studies (Guo et al., 2021; Bohnsack et al., 2019; Delaunay and Frye, 2019); these regulators included 7 “writers” (NOP2, NSUN2, NUSN3, NSUN4, NSUN5, NSUN6, and NSUN7), 1 “reader” (YBX1), and 2 “erasers” (TET2 and TET3). The expression profile of these regulators was systematically extracted and analyzed in normal and tumor samples. The somatic mutation of HCC was assessed with the “maftools” R package. The tumor mutation burden (TMB) was calculated, and the correlation between TMB and clinical characteristics was evaluated. The expression levels of m5C regulators in special immune cells were investigated using the Tumor Immune Single-Cell Hub (TISCH) (http://tisch.comp-genomics.org/) (Sun et al., 2021). A pie plot was used to show the cell number of each cell type. UMAP and violin plots were used to show the expression of m5C regulators in different immune cell types. The prognostic value of the m5C regulators was assessed using the Kaplan–Meier (KM) curve with log-rank test.

### Model-Based Clustering Analysis for m5C Regulators

Based on the expression matrix of m5C regulators, unsupervised clustering was performed to identify distinct m5C modification patterns in HCC patients according to the best cutoff using the “ConsensusClusterPlus” R package, and the stability of clustering was guaranteed by 1,000 repetitions (Wilkerson and Hayes, 2010). The optimal number of clusters was determined by the consensus clustering algorithm. Survival analysis was performed between distinct clusters with the KM method. The differences in the biological processes between the distinct clusters were investigated through gene set variation analysis (GSVA) using the “GSVA” R package. The “c2. cp.kegg.v7.4. symbols” gene set was obtained from the Molecular Signatures Database (MSigDB). An adjusted *p* value <0.05 was considered statistically significant.

### Comparison of the Tumor Immune Microenvironment Between Distinct m5Cclusters

Single-sample gene set enrichment analysis (ssGSEA) was used to quantify the relative infiltration levels of 23 immune cell types in HCC samples (Charoentong et al., 2017). The ratios of the immune stromal components in the tumor microenvironment (TME) were measured using Estimation of Stromal and Immune cells in Malignant Tumor tissues using Expression data (ESTIMATE) analysis with the “estimate” R package (Yoshihara et al., 2013). The differences in the TME between the different clusters were analyzed with the Wilcoxon rank sum test. Furthermore, the “limma” R package was used to investigate the differences in the expression of targeted immune checkpoint molecules between the different clusters.

### Identification of Prognosis-Related Differentially Expressed Genes Between the Distinct m5Cclusters

Principal component analysis (PCA) was used to investigate whether there were different m5C modification patterns in HCC. The empirical Bayesian approach was applied to extract differentially expressed genes (DEGs) between the distinct m5Cclusters. The significance criterion of DEGs was set as an adjusted *p* value <0.001. Gene Ontology (GO) biological process analysis and Kyoto Encyclopedia of Genes and Genomes (KEGG) pathway analysis were performed to investigate the enriched functional annotations of DEGs. A critical value of adjusted *p*-value = 0.05 was selected as the filter criterion. After obtaining the DEGs, univariate Cox regression analysis was performed to identify prognosis-related genes. The significance criterion was set as an adjusted *p* value <0.001 and abs (logFC) > 0.

### Construction of the m5C Gene Signature

To quantify the m5C modification patterns of individual HCC patients, a set of scoring systems (termed “m5Cscore”) was constructed by PCA. Both principal components 1 and 2 were selected to act as signature scores. The m5Cscore was defined using a method similar to the Genomic Grade Index (GGI) (Sotiriou et al., 2006; Zeng et al., 2019):

m5Cscore=∑(PC1 i +PC2 i)

where i is the expression of overlapping genes with significant prognosis-related DEGs among the m5Cclusters. The m5Cscore was calculated in both the TCGA and ICGC cohorts.

According to the score, samples were divided into high- and low-m5Cscore groups. Correlation analyses were performed to investigate the relationships between the m5Cscore and some related biological pathways, including (Bray et al., 2018) survival analysis, (Islami et al., 2021), immunocorrelation analysis, (Bosetti et al., 2014), clinical correlation analysis, (Hester et al., 2020), TMB, and (Sayiner et al., 2017) targeted immune checkpoint molecules.

### The Human Protein Atlas

The immunohistochemistry (IHC) results showing the protein expression of m5C regulators were downloaded from The Human Protein Atlas (HPA) website (https://www.proteinatlas.org/). The corresponding patient information, staining, intensity and quantity were obtained online (Supplementary Table S1).

### RNA Isolation and Quantitative Real-Time PCR

Total RNA from 10 HCC samples and 10 adjacent tissues was extracted using the FastPure^®^ Cell/Tissue Total RNA Isolation Kit V2 (Vazyme, RC112-01) according to the manufacturer’s instructions. The total RNA concentration and purity were detected by a Nanodrop 2000 spectrophotometer (Thermo Fisher, United States). Samples were then reverse-transcribed into cDNA with HiScript^®^ III RT SuperMix for qPCR (Vazyme, RC323-01) according to the manufacturer’s protocol. qRT–PCR analysis was performed using ChamQ Universal SYBR qPCR Master Mix (Vazyme, Q711-02/03) on a QuantStudio 3 (Applied Biosystems, United States) to measure the expression levels of m5C regulators. The expression levels of the gene were normalized to β-actin and analyzed by the 2−ΔΔCt method. The primers used in this study are shown in Supplementary Table S2.

### Statistical Analysis

All statistical analyses were performed with R software (version 4.0.5) and GraphPad Prism software (version 8.3.0). Paired t tests were performed to compare the expression levels of m5C regulators in HCC tissues. Continuous variables were dichotomized for patient survival using the optimal cutoff values determined by the “survminer” R package. The survival curves for the prognostic analysis were constructed by the KM method, and log-rank tests were used to identify the significance of differences. Receiver operating characteristic (ROC) curves (R package “timeROC”) and the area under the curve (AUC) values were used to evaluate the prognostic value of the m5Cscore (Blanche et al., 2013). Univariate and multivariate independent prognostic analyses were performed to assess whether the model was an independent prognostic factor for HCC. All statistical *p* values were two-sided, with *p* < 0.05 deemed statistically significant.

## Results

### Expression Variation of the m5C Regulators in Hepatocellular Carcinoma

**FIGURE 1:**
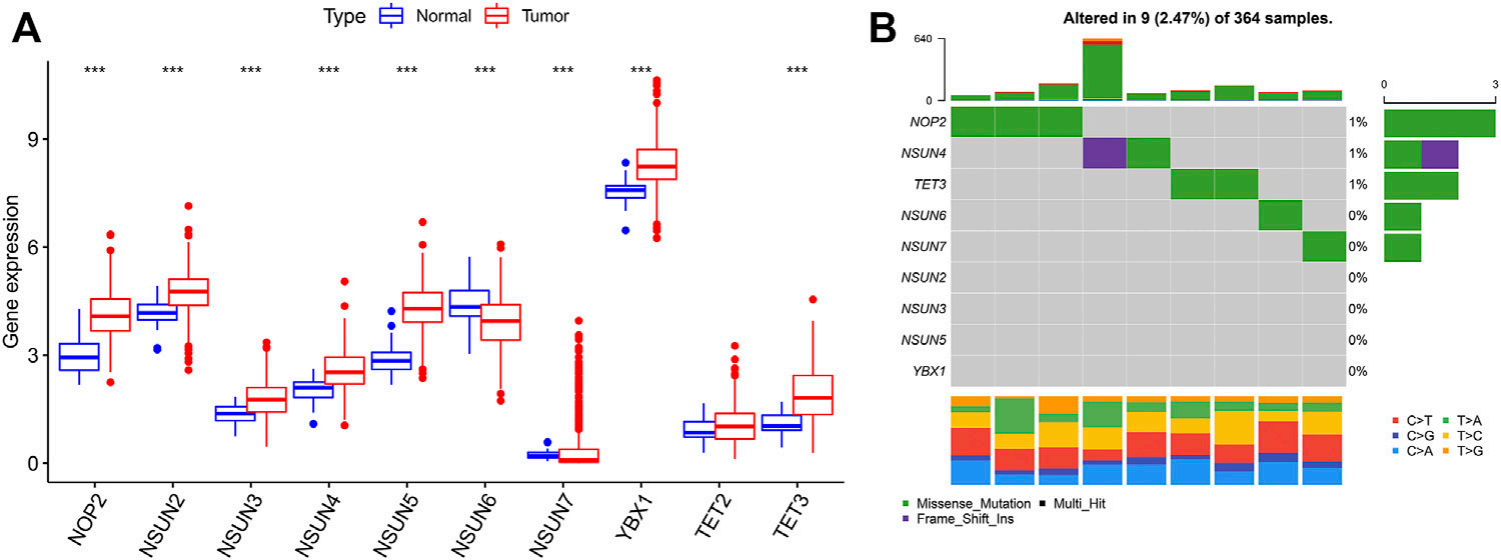
5C modification pattern in HCC. **(A)** The expression of m5C regulators in tumor and normal tissues; **(B)** The mutation frequency of m5C regulators in HCC (Each column represents a patient with a m5C regulator mutation, and the upper panel shows the tumor mutation burden. 0–3 means the number of patients with mutation, and 0–640 means the total mutation frequency of each patient).

In this study, 374 HCC and 50 normal samples in the TCGA cohort were analyzed. The results revealed that almost all enrolled m5C regulators were differentially expressed between HCC and normal tissues, while there was no difference in the expression of TET2 (Figure 1A). Most m5C regulators were upregulated in HCC tissues (*p* < 0.001), while the expression levels of NSUN6 and NSUN7 were downregulated in HCC tissues. Somatic mutations were investigated to explore the prevalence of m5C regulator variations in HCC. The overall average mutation frequency of m5C regulators was low, with only 9 of 364 samples having m5C regulator mutations (Figure 1B). The mutation frequency was higher in NOP2 than in other regulators. Furthermore, the TMB was different in HCC patients with different clinicopathological characteristics. Variation analysis showed that patients in the male or N0 stage had a higher TMB, and a similar result could be found in patients older than 65 years (Supplementary Figure S1).

**FIGURE 2:**
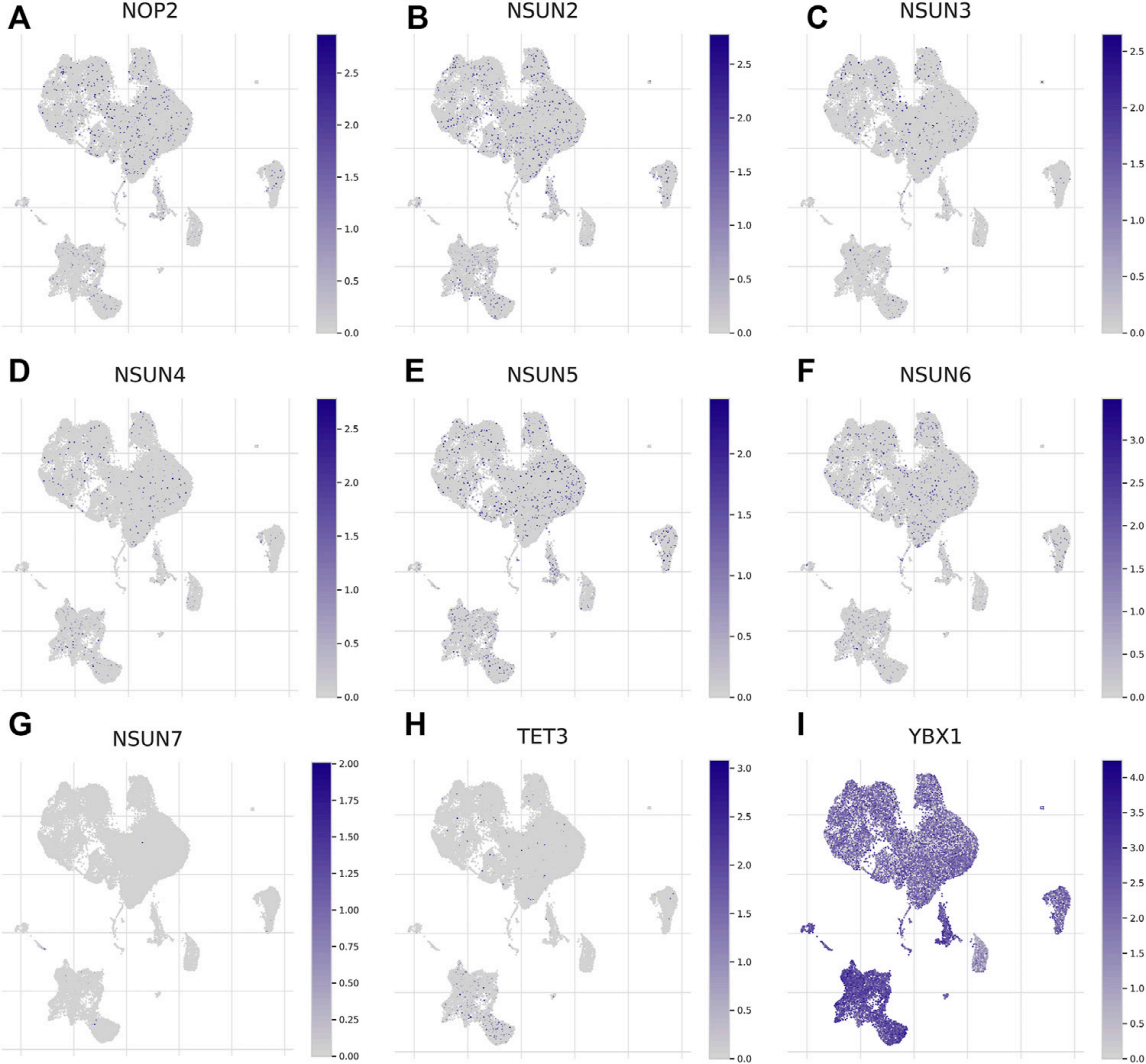
Expression of m5C regulators in tumor microenvironment-related cells (TISCH). **(A-I)** The expression of m5C regulators in immune cells.

Considering the role of the TME in tumor occurrence and progression, we used a data set (GSE140228) in the TISCH database to analyze the expression levels of m5C regulators in TME-related cells. As shown in Supplementary Figure S2, GSE140228 was divided into 20 cell clusters and 12 types of cells, and CD8^+^ T cells were the most abundant immune cells (*n* = 19969). The expression level of YBX1 was highest in TME-related cells, while NSUN7 and TET3 were hardly expressed (Figures 2A–I). In addition, the expression levels of m5C regulators were different in distinct immune cells (Supplementary Figure S3).

**FIGURE 3:**
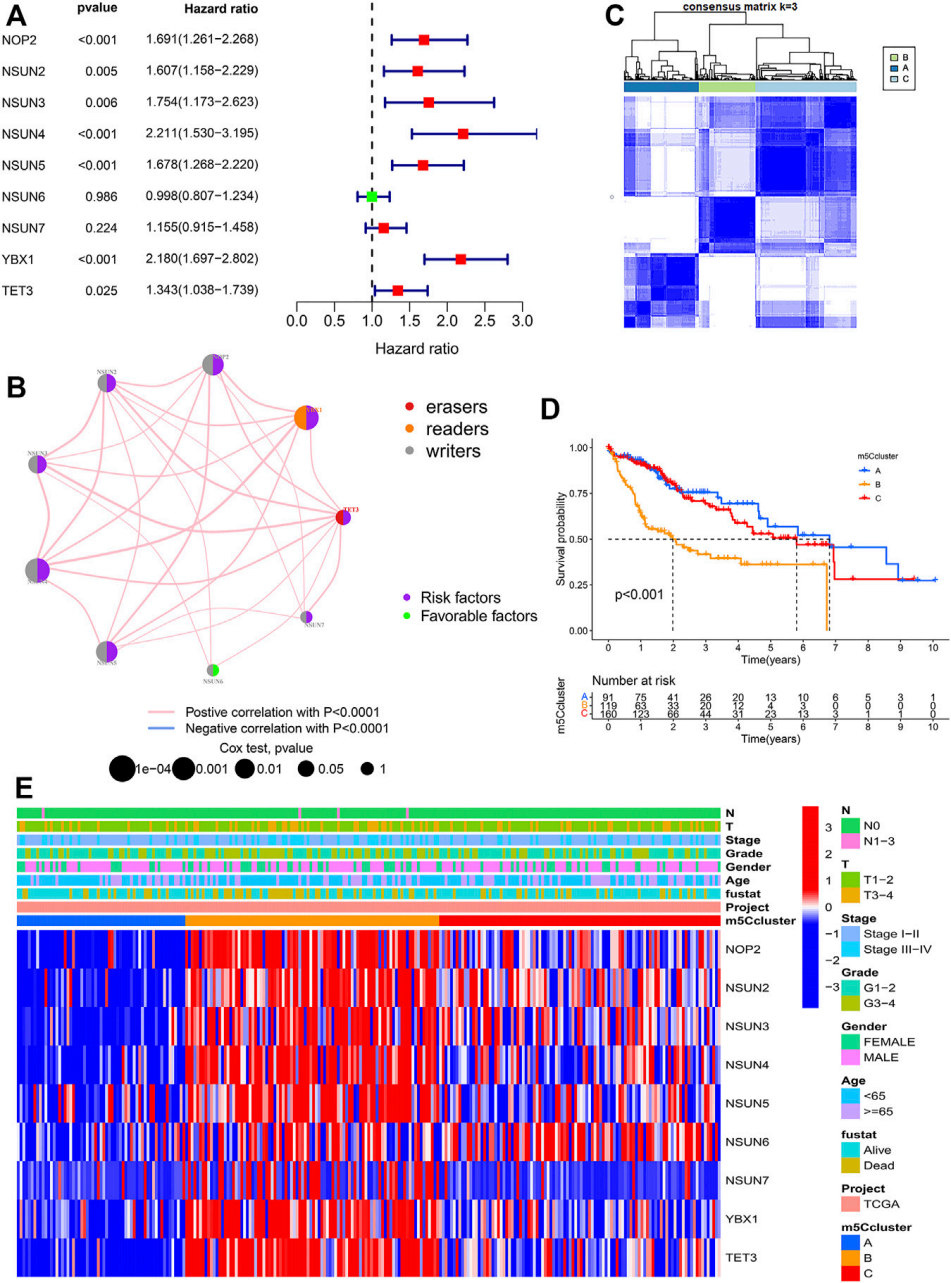
Molecular characteristics of m5Cclusters. **(A)** Forest plot of univariate Cox regression analysis results; **(B)** Interaction of m5C regulators in HCC; **(C)** Consensus clustering matrix for k = 3; **(D)** Survival analysis of patients in distinct m5Cclusters; **(E)** Heatmap depicting the expression levels of m5C regulators in distinct m5Cclusters.

Univariate Cox regression analysis and the KM method showed that most m5C regulators were potential prognostic risk factors for HCC patients (Supplementary Table S3, Figure 3A, Supplementary Figure S4). The network of m5C regulators comprehensively demonstrated the interactions, connections, and prognostic significance of m5C regulators in HCC patients (Figure 3B). The results showed that there were distinct positive correlations between each other. Most regulators, such as NSUN4 and YBX1, presented tumorigenic characteristics, with higher gene expression levels correlated with poor prognosis. Conversely, NSUN6 presented tumor-suppressing characteristics, with higher gene expression levels related to favorable prognosis. Overall, the above results presented high heterogeneity of the genome and expression variations of m5C regulators between normal and HCC tissues, indicating that m5C regulators may play a crucial role in HCC occurrence and progression.

### m5C Modification Patterns Mediated by m5C Regulators

Based on the expression of the 9 m5C regulators, model-based clustering was performed to classify HCC patients using the “ConsensusClusterPlus” R package. Through unsupervised clustering, three distinct m5C modification patterns were ultimately uncovered (identified as m5Cclusters A-C), including 91 cases in cluster A, 119 cases in cluster B, and 160 cases in cluster C (Figure 3C). Prognostic analysis showed that there was a survival advantage in cluster A and a survival disadvantage in cluster B (Figure 3D). Further analysis revealed that there was a significant difference in three distinct m5C modification patterns. m5Ccluster A presented significantly low expression of all m5C regulators, while m5Ccluster B was characterized by high expression of all regulators (Figure 3E). Therefore, it was not surprising that m5Ccluster B had the poorest prognosis. In addition, GSVA was performed to investigate the differences in the biological process among the distinct m5Cclusters. The results indicated that distinct m5C modifications had a significant effect on the biological behaviors of HCC (Supplementary Figure S5).

### Tumor Immune Characteristics in Distinct m5C Modification Patterns

**FIGURE 4:**
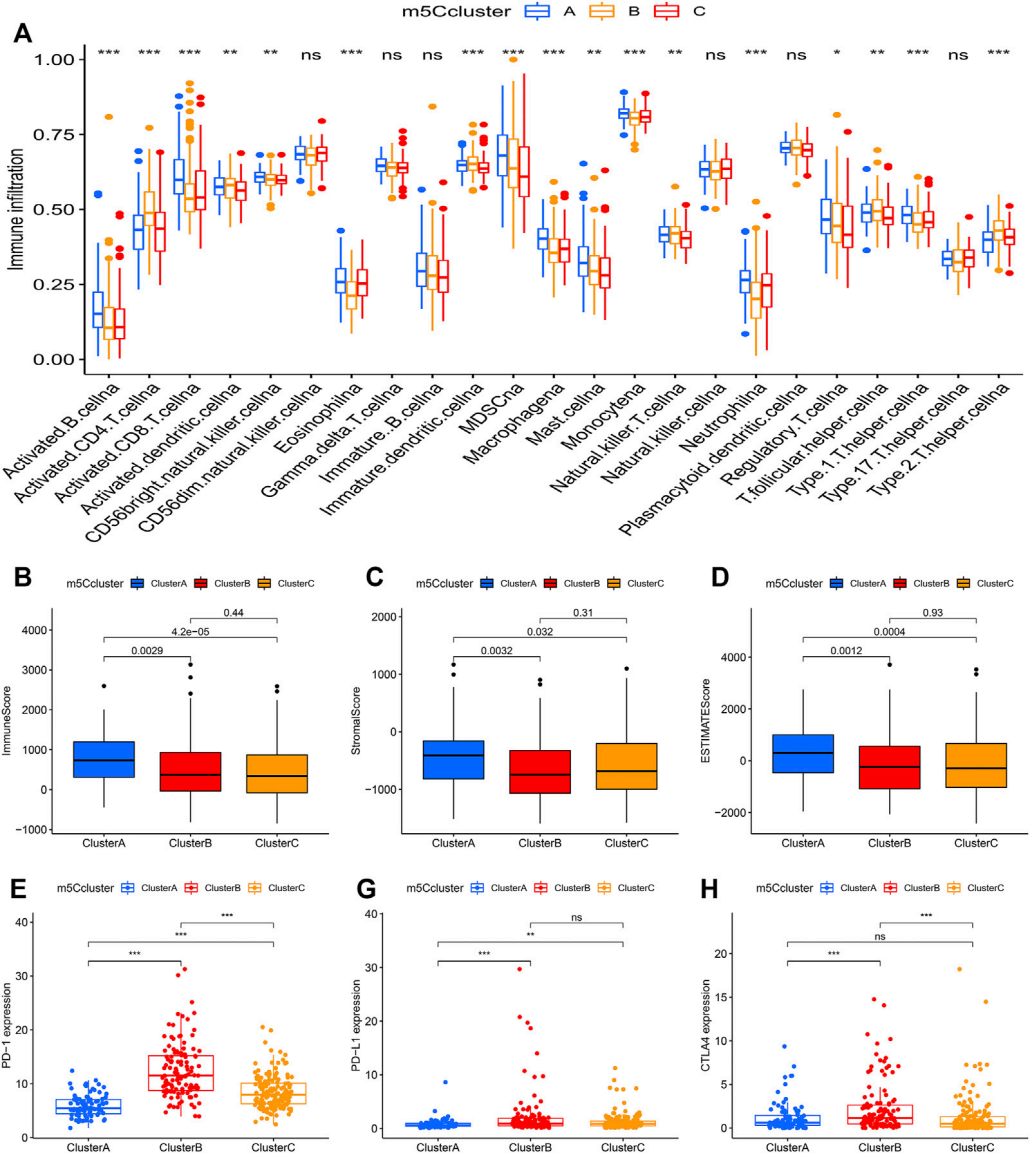
Tumor immune landscape in distinct m5Cclusters. **(A)** ssGSEA of patients in distinct m5Cclusters, the asterisks represent the statistical *p* value between the three m5Cclusters (*p < 0.05; **p < 0.01; ***p < 0.001); **(B)** Immune score of patients in distinct m5Cclusters; **(C)** Stromal score of patients in distinct m5Cclusters; **(D)** ESTIMATE score of patients in distinct m5Cclusters; **(E)** PD-1 expression in distinct m5Cclusters (*p < 0.05; **p < 0.01; ***p < 0.001); **(F)** PD-L1 expression in distinct m5Cclusters (*p < 0.05; **p < 0.01; ***p < 0.001); **(G)** CTLA-4 expression in distinct m5Cclusters (*p < 0.05; **p < 0.01; ***p < 0.001).

Through ssGSEA, the difference in the infiltration of 23 different immune cell types was assessed in the distinct m5Cclusters (Figure 4A). m5Ccluster A showed high infiltration of activated B cells, activated CD8^+^ T cells, eosinophils and monocytes, while m5Ccluster B was characterized by high infiltration of activated CD4^+^ T cells and T helper type 2 (Th2) cells. In addition, the results of the ESTIMATE algorithm revealed that the immune, stromal, and ESTIMATE scores (*p* < 0.05) were higher in cluster A than in clusters B and C, while there was no difference between clusters B and C (Figures 4B–D). Meanwhile, the expression of targeted immune checkpoint molecules was different among the distinct clusters. The boxplots showed that the expression of the PD-1, PD-L1 and CTLA-4 genes was markedly higher in cluster B and significantly lower in cluster A (Figures 4E–G). It was surprising that the expression levels of targeted immune checkpoint molecules showed a similar trend with the expression levels of the m5C regulators. Characterized by high expression levels of the m5C regulators, m5Ccluster B also had high expression levels of the targeted immune checkpoint molecules.

### Generation of the m5Cscore Model

**FIGURE 5:**
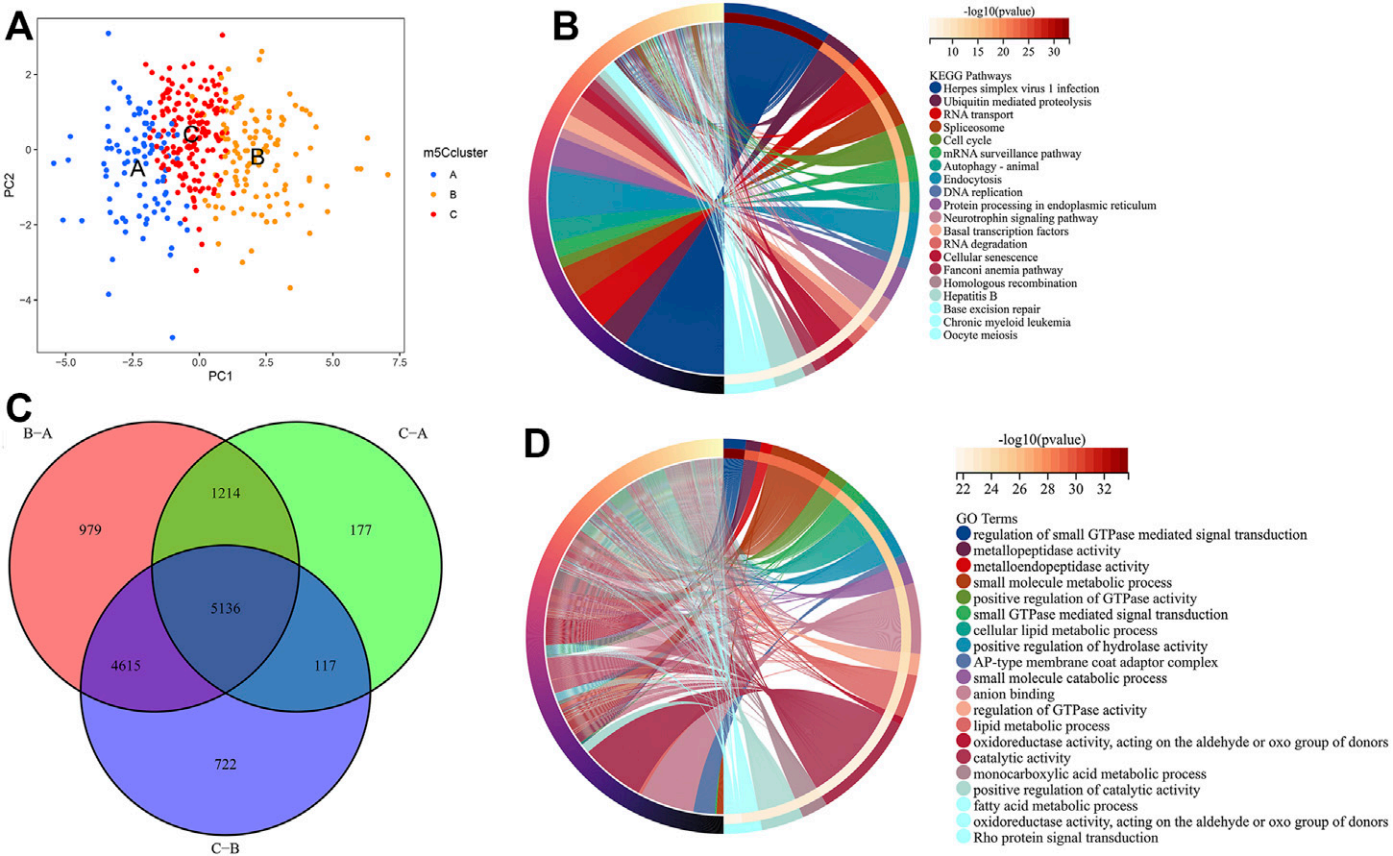
Molecular characteristics of differentially expressed genes (DEGs) among m5Cclusters. **(A)** PCA among distinct m5Cclusters; **(B)** DEGs extracted among three m5Cclusters; **(C)** KEGG pathway analysis for the DEGs; **(D)** GO enrichment analysis for the DEGs.

PCA indicated that there were distinct m5C modification patterns in HCC (Figure 5A). To further investigate the potential biological behavior of each m5Ccluster, a total of 5,136 DEGs were extracted from the distinct m5Cclusters (Figure 5B). GO enrichment analysis and KEGG pathway analysis were performed with the “clusterProfiler” R package. The results showed that the DEGs were enriched in biological processes related to tumorigenesis and tumor progression, such as the cell cycle and autophagy (Figures 5C,D).

**FIGURE 6:**
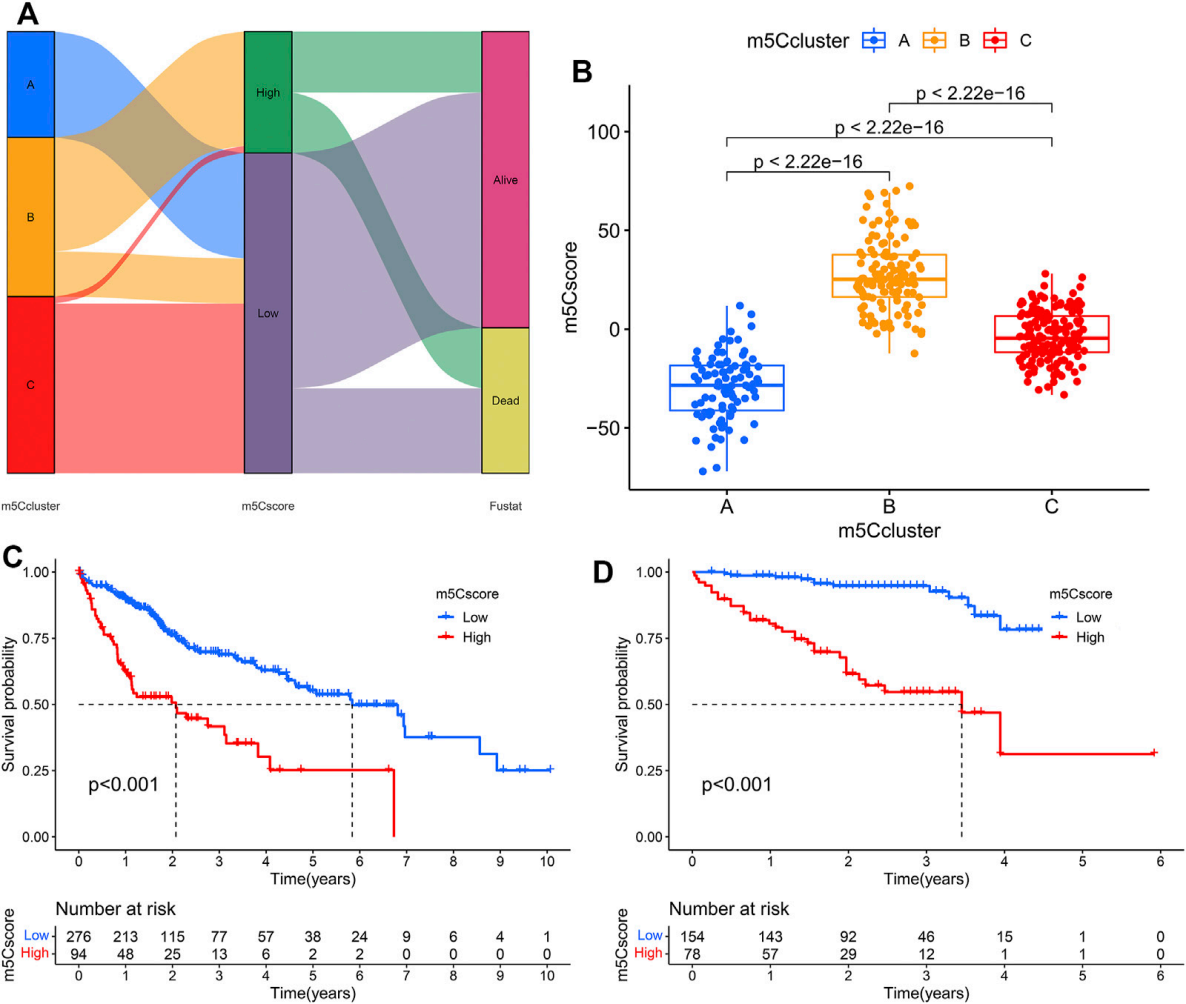
Construction of the m5Cscore model. **(A)** Alluvial diagram showing the changes in m5Cclusters and m5Cscore; **(B)** Differences in m5Cscore among the three m5Cclusters; **(C)** KM analysis of patients in the high- and low-m5Cscore groups (TCGA cohort); **(D)** KM analysis of patients in the high- and low-m5Cscore groups (ICGC cohort).

Univariate Cox regression analysis was performed to investigate the prognostic value of each DEG, and 1,183 genes with prognostic utility were eventually extracted to construct the patients’ individual m5Cscore. The best cutoff value was calculated, and the patients were divided into low- and high-m5Cscore groups. An alluvial diagram was used to visualize the changes in the attributes of individual HCC patients and showed that m5Ccluster B was linked to a high m5Cscore and had the highest proportion of deaths (Figure 6A). Furthermore, the relationship between m5C modification and the m5Cscore was explored. Differential analysis found that m5Ccluster B had the highest m5Cscore, while m5Ccluster A had the lowest m5Cscore (Figure 6B).

**FIGURE 7:**
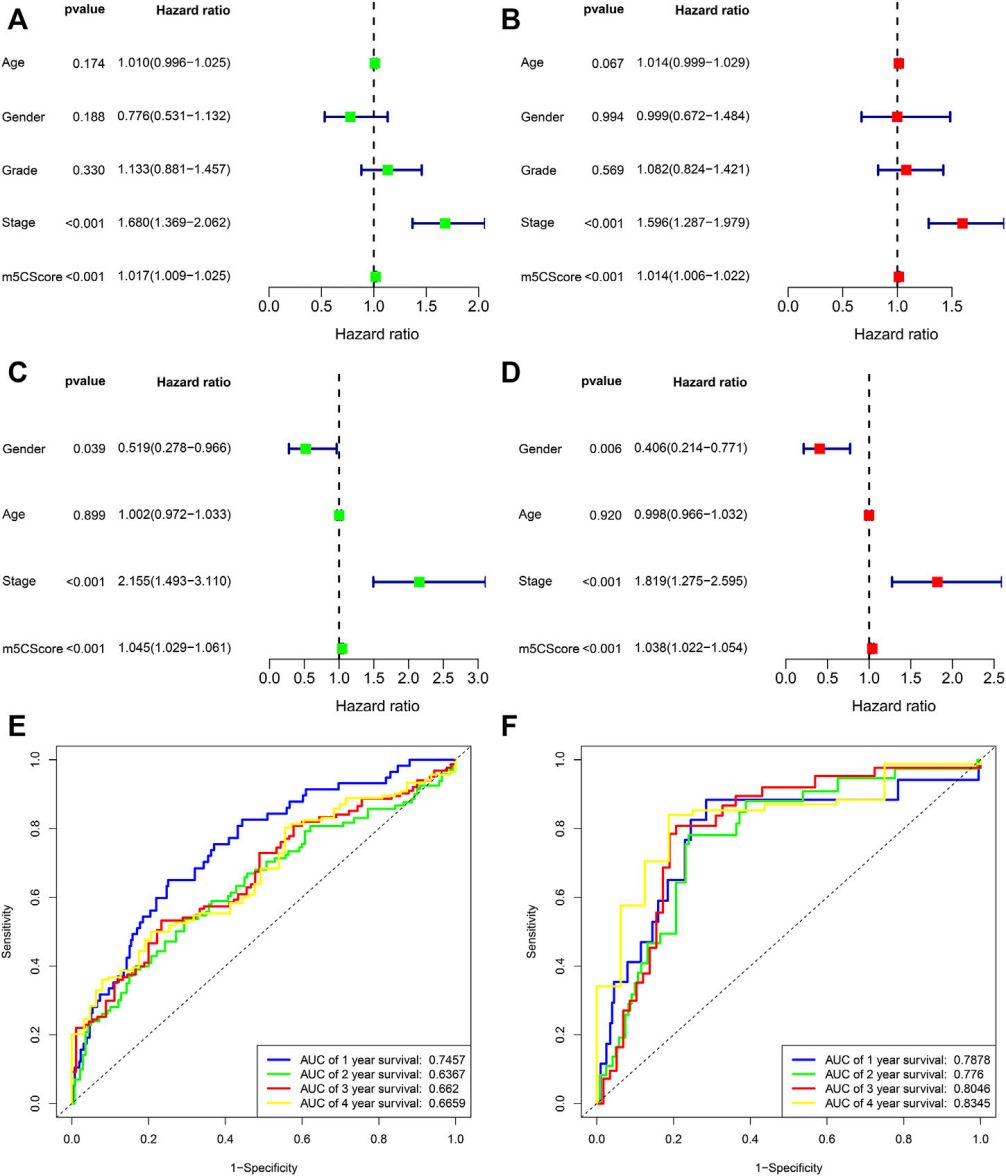
Prognostic value of the m5Cscore model. **(A)** Univariate independent prognostic analysis in TCGA cohort; **(B)** Multivariate independent prognostic analysis in TCGA cohort; **(C)** Univariate independent prognostic analysis in ICGC cohort; **(D)** Multivariate independent prognostic analysis in ICGC cohort; **(E)** Receiver operating characteristic (ROC) curves of m5Cscore for predicting the 1/2/3/4/5-years survival in TCGA cohort; **(F)** Receiver operating characteristic (ROC) curves of m5Cscore for predicting the 1/2/3/4/5-years survival in ICGC cohort.

Patients with low m5Cscores demonstrated a prominent survival benefit in both the TCGA and ICGC cohorts (Figures 6C,D). In addition, univariate and multivariate Cox regression analyses including sex, age, tumor grade, m5Cscore, and tumor stage were performed in the TCGA and ICGC cohorts, which confirmed that m5Cscore was an independent prognostic factor of HCC (in TCGA cohort: HR: 1.045, 95% CI: 1.029–1.061, *p* < 0.001; in ICGC cohort: HR: 1.038, 95% CI: 1.022–1.054, *p* < 0.001, respectively) (Figures 7A–D). The AUC curves indicated that the m5Cscore had an acceptable prognostic value for HCC patients. The AUC values for predicting 1-, 2-, 3-, and 4-years OS in the TCGA cohort were 0.75, 0.64, 0.66, and 0.67, respectively, and those in the ICGC cohort were 0.79, 0.78, 0.80, and 0.83, respectively (Figures 7E,F).

**FIGURE 8:**
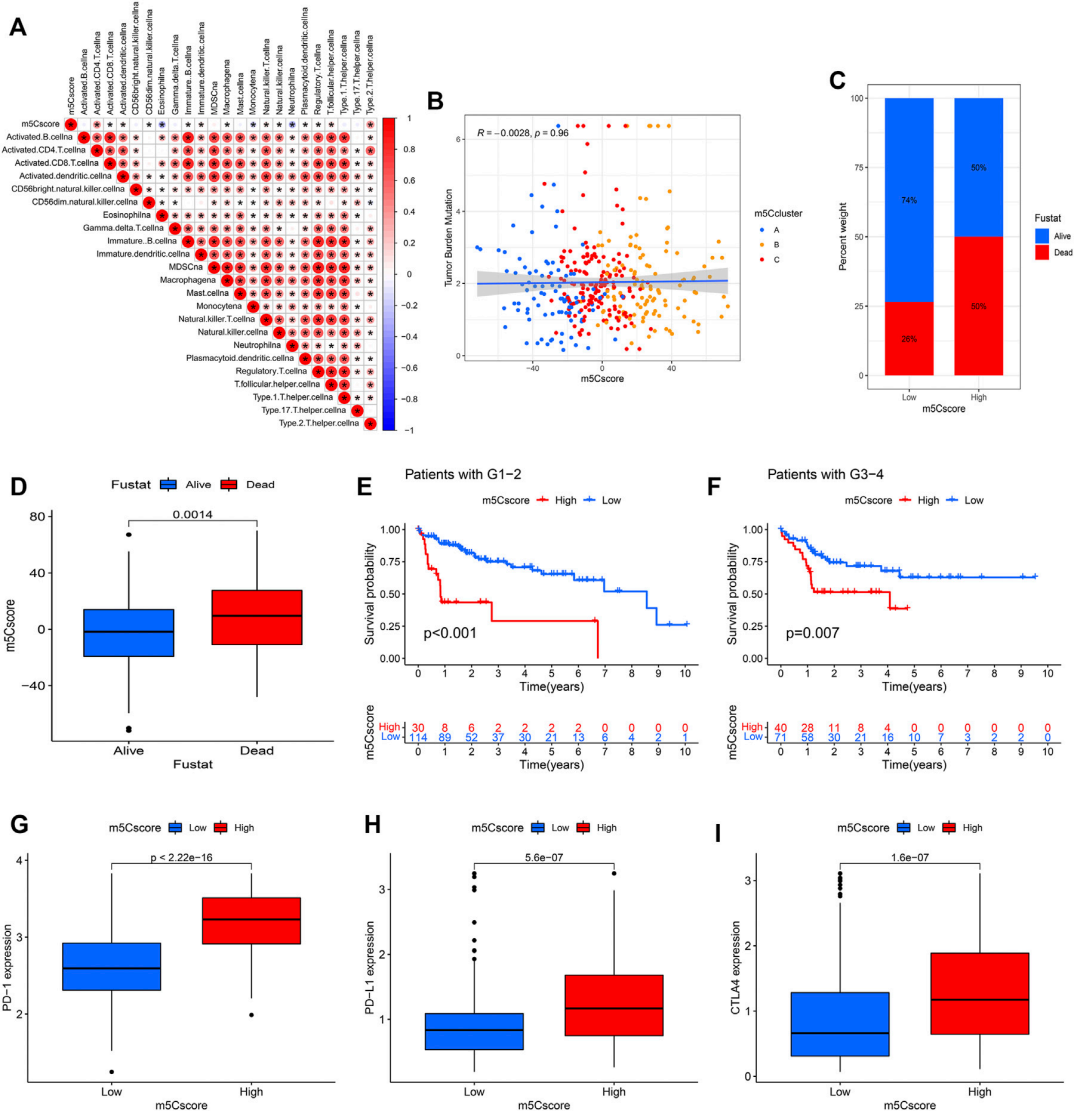
Correlation analysis between the m5Cscore and some related biological pathways. **(A)** Immunocorrelation analysis; **(B)** Tumor mutation burden (TMB) correlation analysis; **(C,D)** Clinical correlation analysis; **(E,F)** Model validation in patients with different tumor grades; **(G)** PD-1 expression in distinct m5Cscore groups; **(H)** PD-L1 expression in distinct m5Cscore groups; **(I)** CTLA-4 expression in distinct m5Cscore groups.

To investigate the potential biological mechanism of the m5Cscore, we analyzed the correlations between the m5Cscore and some biological processes. As shown in Figure 8A, there were significantly positive correlations between the m5Cscore and some infiltrated immune cells, such as activated CD4^+^ T cells and Th2 cells, while there was a negative correlation between the m5Cscore and the infiltration of eosinophils, monocytes and neutrophils. Unfortunately, there was no significant relationship between TMB and the m5Cscore (*p* = 0.96) (Figure 8B). In addition, patients with high m5Cscores had a high proportion of deaths (Figures 8C,D). Furthermore, the model validation results indicated that the m5Cscore model could be suitable for patients with different tumor grades (Figures 8E,F). Immunotherapies involving PD-1, PD-L1 and CTLA-4 blockade have undoubtedly emerged as a major breakthrough in cancer therapy. Patients with high m5Cscores showed obviously high expression levels of PD-1, PD-L1, and CTLA-4, which indicated a potential response to anti-PD-1/PD-L1/CTLA-4 therapy (Figures 8G–I).

### The mRNA and Protein Expression Levels of m5C Regulators in Hepatocellular Carcinoma

**FIGURE 9:**
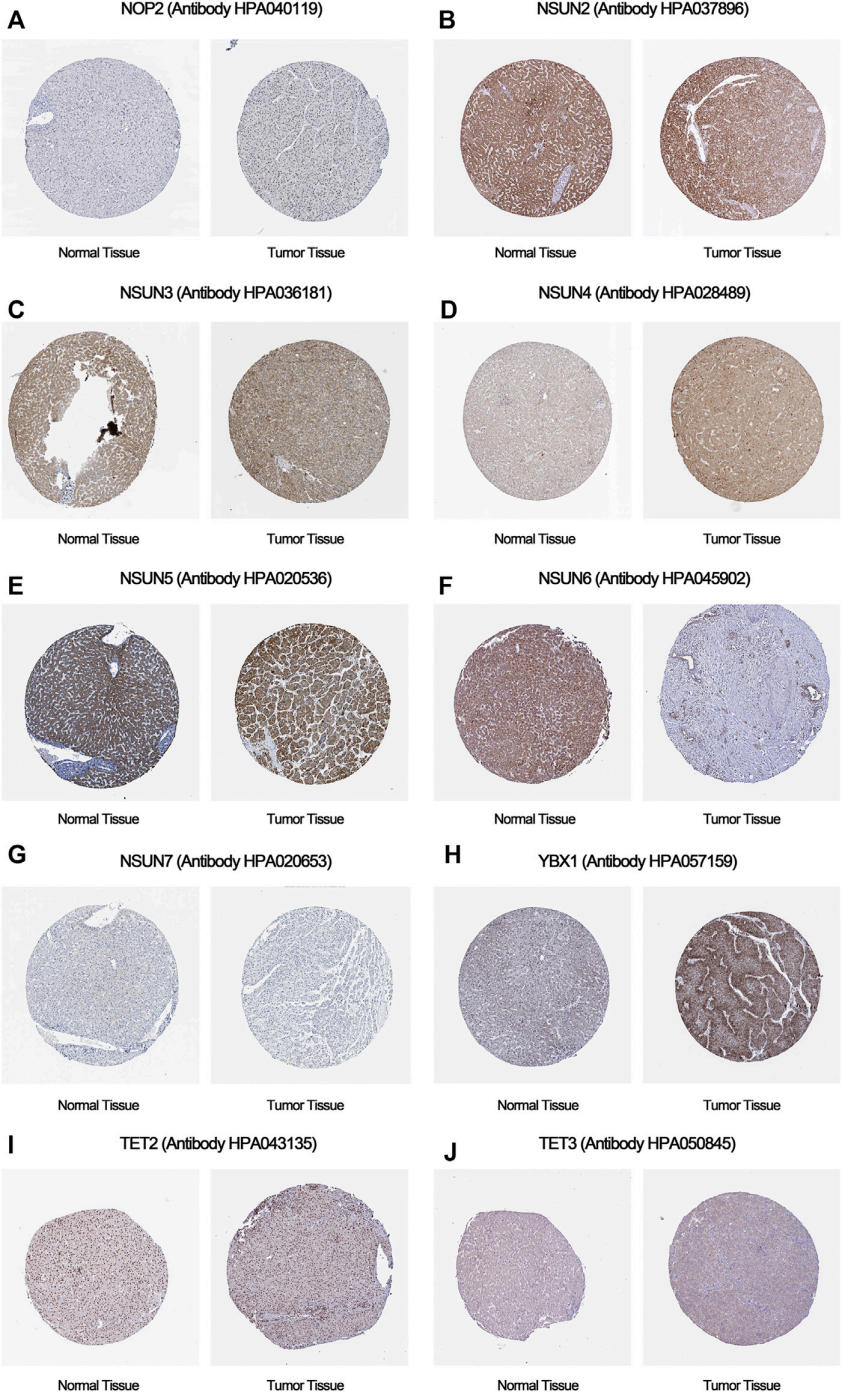
The protein expression of m5C regulators in HCC and normal tissues. **(A)** The protein expression of NOP2 in HCC and normal tissues; **(B)** The protein expression of NSUN2 in HCC and normal tissues; **(C)** The protein expression of NSUN3 in HCC and normal tissues; **(D)** The protein expression of NSUN4 in HCC and normal tissues; **(E)** The protein expression of NSUN5 in HCC and normal tissues; **(F)** The protein expression of NSUN6 in HCC and normal tissues; **(G)** The protein expression of NSUN7 in HCC and normal tissues; **(H)** The protein expression of YBX1 in HCC and normal tissues; **(I)** The protein expression of TET2 in HCC and normal tissues; **(J)** The protein expression of TET3 in HCC and normal tissues.

**FIGURE 10:**
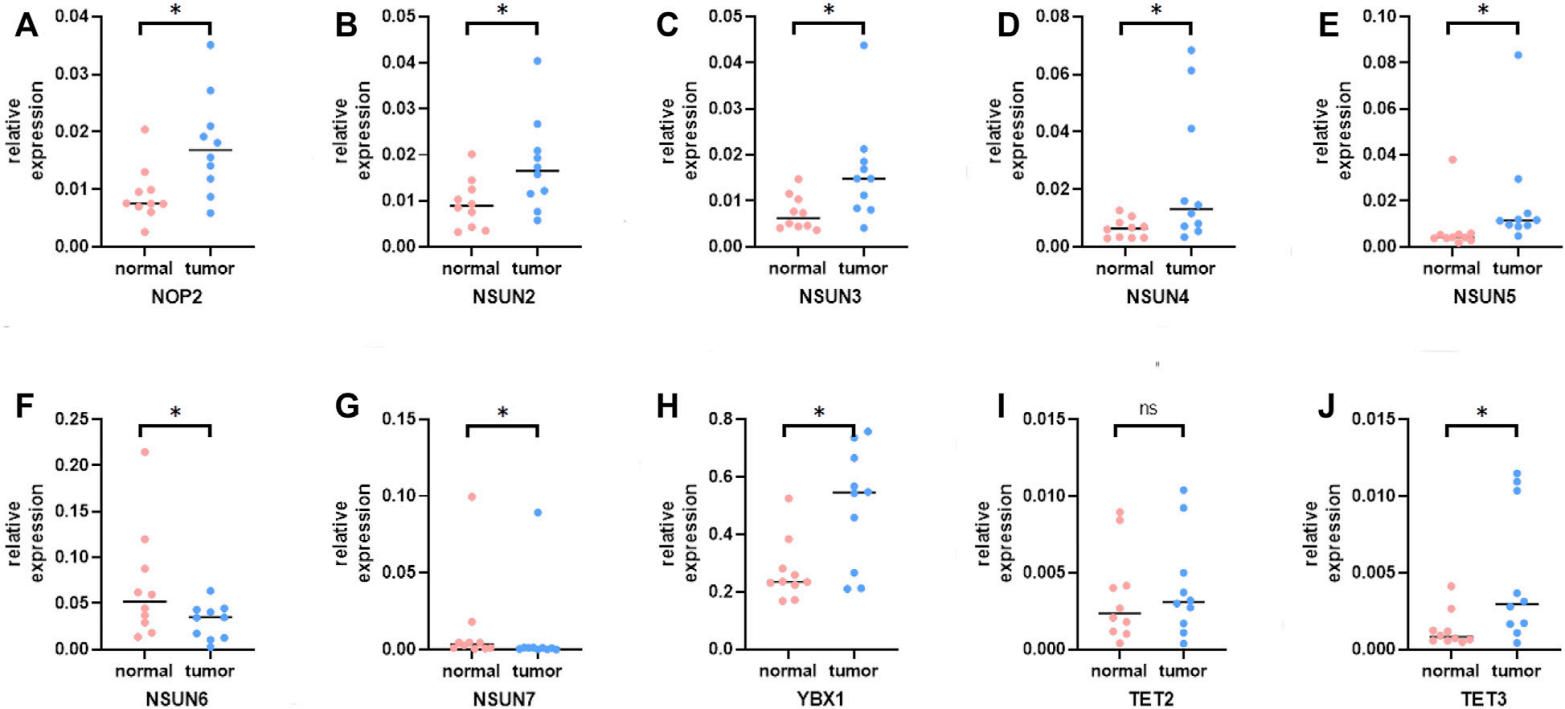
The mRNA expression of m5C regulators in HCC and normal tissues. **(A)** The mRNA expression of NOP2 in HCC and normal tissues; **(B)** The mRNA expression of NSUN2 in HCC and normal tissues; **(C)** The mRNA expression of NSUN3 in HCC and normal tissues; **(D)** The mRNA expression of NSUN4 in HCC and normal tissues; **(E)** The mRNA expression of NSUN5 in HCC and normal tissues; **(F)** The mRNA expression of NSUN6 in HCC and normal tissues; **(G)** The mRNA expression of NSUN7 in HCC and normal tissues; **(H)** The mRNA expression of YBX1 in HCC and normal tissues; **(I)** The mRNA expression of TET2 in HCC and normal tissues; **(J)** The mRNA expression of TET3 in HCC and normal tissues.

To further verify the trend of m5C regulator expression in HCC tissues, we performed a qPCR assay and acquired IHC pathological specimen data from the HPA. As shown in Figures 9, 10, almost all m5C regulators were differentially expressed between HCC and normal tissues. Most m5C regulators were upregulated in HCC tissues, while the expression levels of NSUN6 and NSUN7 were significantly downregulated in HCC tissues.

## Discussion

Multiple m^5^C regulators have been identified as participants in the development and progression of cancer. Specifically, m^5^C plays an important role in cancer cell proliferation and metastasis, as well as cancer stem cell development, by regulating mRNA stability, expression, and translation (Lu et al., 2018; Shen et al., 2018; Chen et al., 2019; Mei et al., 2020). For instance, Ban et al. (Ban et al., 2021) found that YBX1 could promote nasopharyngeal carcinoma (NPC) cell proliferation and invasiveness by enhancing the protein synthesis of AURKA. As one of the most widespread malignances worldwide, HCC remains poorly understood in terms of its pathogenesis and development (Xue et al., 2020). Recently, the role of m5C modification in tumors has attracted great attention, and increasing evidence has revealed that m5C modification is closely related to the tumorigenesis and tumor progression of HCC (He et al., 2020b; He et al., 2020c; Zhang et al., 2020). Sun et al. (Sun et al., 2020) found that the NSUN2-mediated m^5^C modification of H19 lncRNA exerts an important function in the progression and malignancy of HCC. However, most of these studies focused on a single m5C regulator or only explored the distribution of m5C in HCC, and the overall influence of m5C regulator-related modification patterns on tumor prognosis has not been fully established.

In this study, we demonstrated that m5C modification played a crucial role in the tumorigenesis and tumor progression of HCC and had potential prognostic value for HCC. To clarify the role of m5C in HCC, we comprehensively profiled the m5C modification patterns in HCC samples obtained from public databases. Through unsupervised clustering analyses, we identified three distinct m5C modification patterns in HCC, characterized by differences in prognosis, immune cell infiltration, and pathway signatures. In addition, to quantify the m5C modifications of individual patients, we constructed a model (termed “m5Cscore”), which was proven to be an independent prognostic factor of HCC. Our results indicated that m5C modification was different in HCC patients and could be a novel prognostic biomarker.

The expression levels of almost all m5C regulators were significantly higher in HCC tissues than in adjacent tissues, and a previous study showed that the degree of mRNA methylation in HCC was significantly higher than that in adjacent tissues (Zhang et al., 2020). These results suggested that the alteration of m5C modification was correlated with the pathogenesis of HCC. As shown in Figure 3D, three distinct m5C modification patterns identified by unsupervised clustering analyses had different survival outcomes. m5Ccluster A, which was characterized by low expression levels of m5C regulators, had a survival advantage, while m5Ccluster B, which presented significantly high expression of m5C regulators, had a survival disadvantage. The results indicated that the expression levels of m5C regulators were closely related to the tumor progression of HCC. Considering the heterogeneity of m5C modification, the m5Cscore model was constructed to quantify the m5C modification patterns of individual HCC patients. Through a comprehensive analysis and validation with the training cohort (TCGA) and validation cohort (ICGC), the m5Cscore was identified as a robust and independent prognostic factor of HCC. In addition, subsequent analysis found that the m5Cscore was closely related to immune cell infiltration. The m5Cscore had a significantly positive correlation with the infiltration of activated CD4^+^ T cells and Th2 cells and a negative correlation with the infiltration of eosinophils, monocytes and neutrophils.

The immune environment that surrounds cancer tissues can affect tumor cell growth and metastasis (Ostrand-Rosenberg, 2008). The different types of immune cells have the potential to either promote or delay tumor development and progression. In this study, we investigated the expression levels of m5C regulators in TME-related cells and found that they were different in distinct immune cells. The results indicated that m5C regulators were closely related to the TME in HCC. In addition, we quantified the infiltration of 23 different immune cell types in HCC samples through ssGSEA. Subsequent analysis found that there was a significant difference in immune cell infiltration among the distinct m5Cclusters. Patients in m5Ccluster A showed high infiltrations of activated B cells, activated CD8^+^ T cells, eosinophils and monocytes, while those in m5Ccluster B were characterized by high infiltrations of activated CD4^+^ T cells and Th2 cells. Renata et al. (Rossetti et al., 2018) demonstrated that activated B cells could induce antigen specific T cell responses and play an antitumor role. Similarly, activated CD8^+^ T cells and eosinophils can inhibit tumor growth in different ways (Wieckowski et al., 2009; Grisaru et al., 2021). In addition, previous research showed that a high number of Th2 cells was associated with poor prognosis in tumors (De Monte et al., 2011). Therefore, with a high infiltration of antitumor immune cells, patients in m5Ccluster A had a survival advantage, while those in m5Ccluster B characterized by high infiltration of tumor promoting immune cells had a worse outcome.

Immune checkpoint inhibitor (ICI) therapy targeting the PD-1/PD-L1/CTLA-4 pathway has been approved for the treatment of more than 10 cancer types (Callahan et al., 2016; Hoos, 2016). As a chance for cure, ICI therapy has revolutionized cancer treatment (Xie et al., 2021). However, only a portion of patients had an expected response during immune checkpoint blockade therapy (Agdashian et al., 2019). For patients with advanced HCC, the anti-PD-1 agents nivolumab and pembrolizumab demonstrated an objective response rate (ORR) of only 15% in patients who had prior treatment with sorafenib (El-Khoueiry et al., 2017; Zhu et al., 2018). A similar situation has occurred in anti-CTLA-4 therapy (Duffy et al., 2017). Recently, the expression levels of PD-1, PD-L1 and CTLA-4 were identified as predictive biomarkers for immunotherapy response (Ferris et al., 2016; Herbst et al., 2016; Muro et al., 2016; Ott et al., 2017). In this study, we found that the expression levels of these targeted immune checkpoint molecules were different in HCC patients. Patients in m5Ccluster B had higher expression of PD-1, PD-L1 and CTLA-4 than those in m5Ccluster A, and a similar situation showed that the expression levels of PD-1, PD-L1 and CTLA-4 were higher in the high m5Cscore group. The results indicated that those patients would have a better response to ICI therapy. This needs to be further evaluated through experiments.

There were several limitations in this study. First, immune cell infiltration was assessed based on algorithms owing to technical limitations. Second, due to a lack of data, we could not directly explore the difference in the response to immunotherapy between the high- and low-m5Cscore groups. Last, there was no clinical cohort to verify the predictive value of the m5Cscore in HCC; thus, further research based on large cohort prospective clinical trials is needed.

In conclusion, this study comprehensively explored and systematically profiled the expression features of m5C-related regulators in HCC. The m5C modification patterns play a crucial role in the TIME and prognosis of HCC. Our work will enhance the understanding of the tumor immune landscape and provide a practical tool for predicting the prognosis of HCC. This study will help clinicians identify effective indicators for HCC to improve the poor prognosis of this disease.

## Data Availability

The datasets presented in this study can be found in online repositories. The names of the repository/repositories and accession number(s) can be found in the article/Supplementary Material.

## Supplementary Material

Supplementary Figure 1 Variation analysis between TMB and clinical status. **(A)** Variation analysis between TMB and age; **(B)** Variation analysis between TMB and sex; **(C)** Variation analysis between TMB and tumor grade; **(D)** Variation analysis between TMB and tumor stage; **(E)** Variation analysis between TMB and T stage; **(F)** Variation analysis between TMB and N stage.

Supplementary Figure 2 Annotation of all cell types in GSE140228 and the percentage of each type of cell.

Supplementary Figure 3 The expression levels of m5C regulators in HCC tumor microenvironment-related cells.

Supplementary Figure 4 KM analysis of patients with distinct m5C regulator expression. **(A)** KM analysis of patients with distinct NOP2 expression; **(B)** KM analysis of patients with distinct NSUN2 expression; **(C)** KM analysis of patients with distinct NSUN3 expression; **(D)** KM analysis of patients with distinct NSUN4 expression; **(E)** KM analysis of patients with distinct NSUN5 expression; **(F)** KM analysis of patients with distinct TET3 expression; **(G)** KM analysis of patients with distinct YBX1 expression.

Supplementary Figure 5
**.**GSVA enrichment analysis between different m5Cclustters. **(A)** GSVA enrichment analysis between m5Ccluster A and m5Ccluster B; **(B)** GSVA enrichment analysis between m5Ccluster A and m5Ccluster C; **(C)** GSVA enrichment analysis between m5Ccluster B and m5Ccluster C.
