# Supplementary material for: Identification of the Expression Patterns and Potential Prognostic Role of 5-Methylcytosine Regulators in Hepatocellular Carcinoma
Source: Front Cell Dev Biol. 2022 Feb 16;10:842220. doi: 10.3389/fcell.2022.842220 (PMC8888979; doi:10.3389/fcell.2022.842220)
Supplement: Supplementary file 1 [file Table1.DOCX]

Table S1 The protein expression in immunohistochemical specimens of m5C regulators in HCC

| **Gene** | **category** | **Patient id** | **Sex** | **Staining** | **Intensity** | **Quantity** | **Location** |
| --- | --- | --- | --- | --- | --- | --- | --- |
| NOP2 | normal tissue | 2429 | male | low | weak | 75%-25% | nuclear |
| NOP2 | tumor tissue | 3346 | female | medium | moderate | >75% | nuclear |
| NSUN2 | normal tissue | 2429 | male | high | strong | >75% | cytoplasmic/membranous |
| NSUN2 | tumor tissue | 3477 | male | high | strong | >75% | cytoplasmic/membranous |
| NSUN3 | normal tissue | 3222 | female | medium | moderate | >75% | cytoplasmic/membranous |
| NSUN3 | tumor tissue | 2177 | female | medium | moderate | >75% | cytoplasmic/membranous |
| NSUN4 | normal tissue | 3402 | female | not detected | weak | <25% | cytoplasmic/membranous |
| NSUN4 | tumor tissue | 2766 | female | medium | moderate | >75% | cytoplasmic/membranous |
| NSUN5 | normal tissue | 3222 | female | high | strong | >75% | cytoplasmic/membranous |
| NSUN5 | tumor tissue | 2766 | female | high | strong | >75% | cytoplasmic/membranous |
| NSUN6 | normal tissue | 3222 | female | medium | moderate | 75%-25% | none |
| NSUN6 | tumor tissue | 3862 | female | medium | moderate | >75% | cytoplasmic/membranous |
| NSUN7 | normal tissue | 3222 | female | not detected | negative | none | none |
| NSUN7 | tumor tissue | 2556 | male | not detected | negative | none | none |
| YBX1 | normal tissue | 3402 | female | low | moderate | <25% | cytoplasmic/membranous |
| YBX1 | tumor tissue | 4822 | male | high | strong | 75%-25% | cytoplasmic/membranous |
| TET2 | normal tissue | 3402 | female | medium | moderate | >75% | nuclear |
| TET2 | tumor tissue | 3954 | male | medium | moderate | >75% | nuclear |
| TET3 | normal tissue | 2429 | male | not detected | negative | none | none |
| TET3 | tumor tissue | 3196 | male | low | moderate | <25% | cytoplasmic/membranous |
