# Supplementary figures and images for: Identification of the Expression Patterns and Potential Prognostic Role of 5-Methylcytosine Regulators in Hepatocellular Carcinoma
Source: Front Cell Dev Biol. 2022 Feb 16;10:842220. doi: 10.3389/fcell.2022.842220 (PMC8888979; doi:10.3389/fcell.2022.842220)

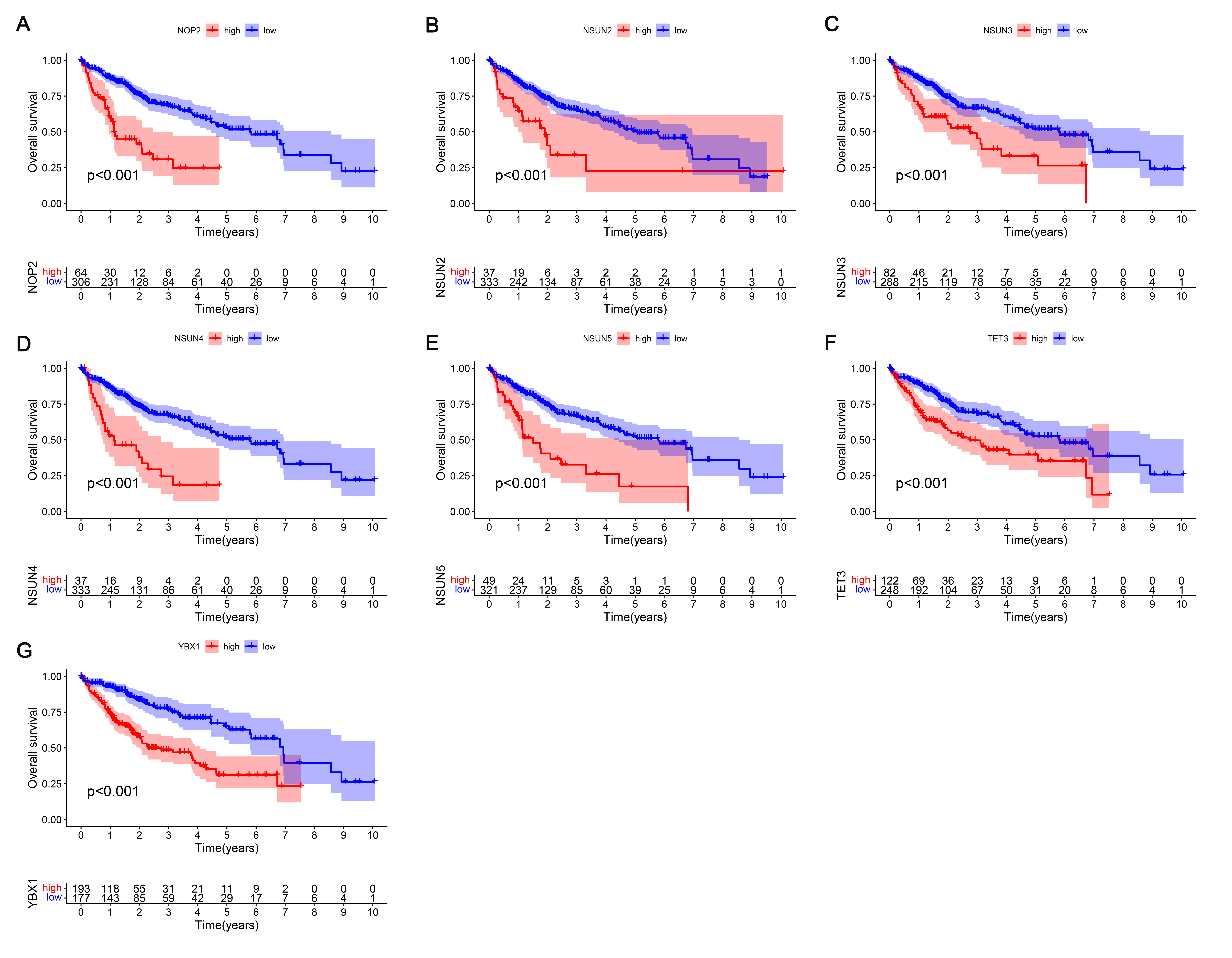

Supplement: Supplementary file 3 [file Image4.TIF]

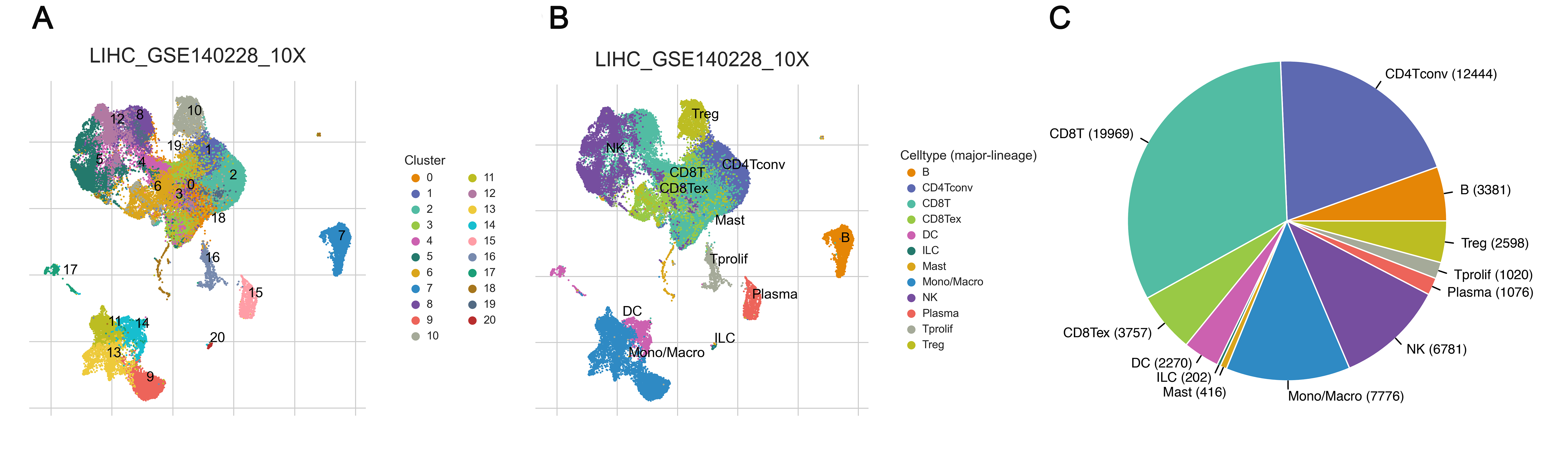

Supplement: Supplementary file 4 [file Image2.TIF]

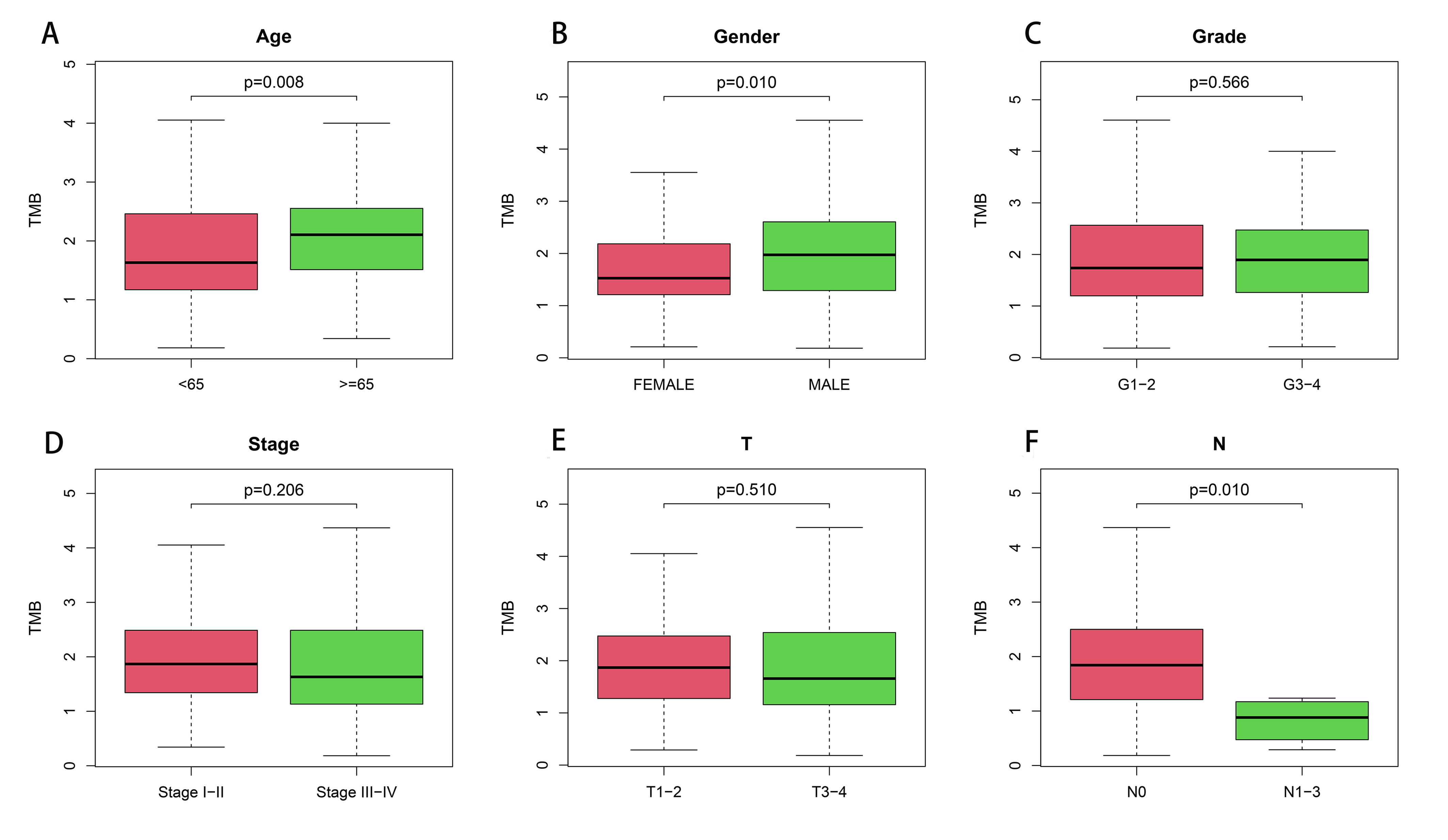

Supplement: Supplementary file 5 [file Image1.TIF]

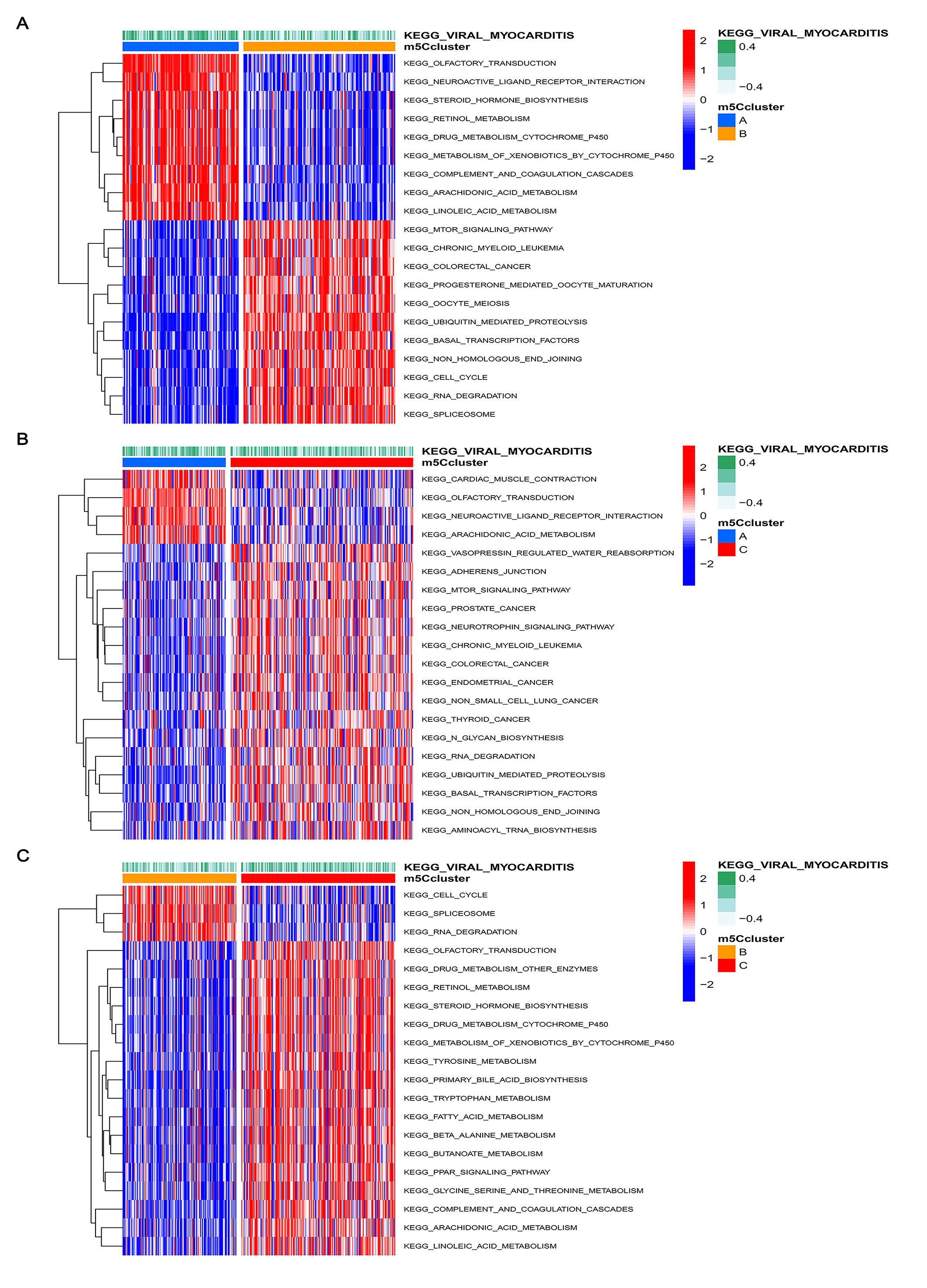

Supplement: Supplementary file 8 [file Image5.TIF]
