# Supplementary material for: Identification of the Expression Patterns and Potential Prognostic Role of 5-Methylcytosine Regulators in Hepatocellular Carcinoma
Source: Front Cell Dev Biol. 2022 Feb 16;10:842220. doi: 10.3389/fcell.2022.842220 (PMC8888979; doi:10.3389/fcell.2022.842220)
Supplement: Supplementary file 6 [file Table2.DOCX]

Table S2 The primer sequences of the m5C regulators

| **gene** | **sequences** |
| --- | --- |
| NOP2 | forward 5 ′ - TGGAGTGAAGAGGAGACCGA-3 ′ |
|  | reverse 5 ′ - TGAACTCGTTGCAGGTCTGG-3 ′ |
| NSUN2 | forward 5 ′ - ACGAAAGATGGGCAGTGGTT-3 ′ |
|  | reverse 5 ′ - AGCTTTTCTGGGTCCTTCGG-3 ′ |
| NSUN3 | forward 5 ′ -TCCAAAGAACTCGGAGATGCC-3 ′ |
|  | reverse 5 ′ -ATCGGTTAAGCAGGACAGCAT-3 ′ |
| NSUN4 | forward 5 ′ - CCAATGATCTCTCCCCGTCC-3 ′ |
|  | reverse 5 ′ - AGTTCTCCCCATTTCCTGCC-3 ′ |
| NSUN5 | forward 5 ′ -CGCCCCAGGCAATAAGAC-3 ′ |
|  | reverse 5 ′ -TGTAGTGGACCTCATGGTAGC-3 ′ |
| NSUN6 | forward 5 ′ -CAGGAACCGCAGATTGGAGG-3 ′ |
|  | reverse 5 ′ -TAATGGCACAGCCGATGGAT-3 ′ |
| NSUN7 | forward 5 ′ -GCTACCTCGTTGTTCAGGACT-3 ′ |
|  | reverse 5 ′ -GCTGTTGAGCAAGAACGTGAA-3 ′ |
| TET2 | forward 5 ′ - GAAGGGTCGAGACAAGGAGC-3 ′ |
|  | reverse 5 ′ - GATGGGATTCCGCTTGGTGA-3 ′ |
| TET3 | forward 5 ′ - CTCATGGAGGAGCGGTATGG-3 ′ |
|  | reverse 5 ′ - CCCTCCTTCCCCGTGTAGAT-3 ′ |
| YBX1 | forward 5 ′ -GTGATGGAGGGTGCTGACAA-3 ′ |
|  | reverse 5 ′ -CCTGCGGAATCGTGGTCTAT-3 ′ |
