# Supplementary material for: Identification of the Expression Patterns and Potential Prognostic Role of 5-Methylcytosine Regulators in Hepatocellular Carcinoma
Source: Front Cell Dev Biol. 2022 Feb 16;10:842220. doi: 10.3389/fcell.2022.842220 (PMC8888979; doi:10.3389/fcell.2022.842220)
Supplement: Supplementary file 7 [file Table3.DOCX]

Table S3 Univariate Cox regression analysis of m5C regulators

| **id** | **HR** | **HR.95L** | **HR.95H** | **P value** | **km** |
| --- | --- | --- | --- | --- | --- |
| NOP2 | 1.69122 | 1.261125 | 2.267995 | 0.000449 | 6.81E-10 |
| NSUN2 | 1.606842 | 1.158252 | 2.229171 | 0.004518 | 0.000964 |
| NSUN3 | 1.753728 | 1.172602 | 2.622852 | 0.006233 | 9.40E-05 |
| NSUN4 | 2.210802 | 1.529798 | 3.194961 | 2.41E-05 | 2.91E-08 |
| NSUN5 | 1.677772 | 1.268085 | 2.219819 | 0.000292 | 3.90E-07 |
| NSUN6 | 0.998111 | 0.80718 | 1.234204 | 0.986072 | 0.137331 |
| NSUN7 | 1.155338 | 0.915478 | 1.458042 | 0.223921 | 0.069649 |
| YBX1 | 2.180409 | 1.696528 | 2.802301 | 1.14E-09 | 6.99E-08 |
| TET3 | 1.34332 | 1.037507 | 1.739274 | 0.025135 | 0.000103 |
